# Evaluation of Peripheral Versus Central Route of Ondansetron as Pretreatment to Prevent Pain on the Injection of Propofol: A Randomized Controlled Study

**DOI:** 10.4274/TJAR.2023.221112

**Published:** 2023-06-16

**Authors:** Deepak Kumar, Prakash K. Dubey, Kunal Singh

**Affiliations:** 1 Department of Anaesthesiology and Critical Care Medicine, Indira Gandhi Institute of Medical Sciences, Patna, India; 2 Department of Anaesthesiology and Critical Care Medicine, All India Institute of Medical Sciences, Patna, India

**Keywords:** Central analgesia, injection pain, local anaesthesia, ondansetron, propofol

## Abstract

**Objective:**

We evaluated whether systemic ondansetron was also useful in the attenuation of propofol injection pain similar to ondansetron pretreatment.

**Methods:**

Eighty patients were enrolled. Patients in group S received ondansetron 4 mg in saline in the right hand followed 30 min later by 5 mL saline in the left hand along with venous occlusion. Group L patients received 4 mL of saline in the right hand followed by 5 mL 4 mg ondansetron in the left hand after 30 min. Two minutes later the occlusion was released. Patients received one-fourth of the calculated total dose of propofol, and their level of pain was graded on a scale of 0 to 3, with 0 denoting no discomfort. Mean blood pressure and heart rates were also recorded. Continuous variables were checked for normality using Shapiro-Wilks test. Normal continuous variables were expressed as mean standard deviation and non-normal continuous variables were expressed as median interquartile range. T-test for the difference in the mean and paired test were used for normally distributed continuous variable whereas Mann-Whitney U test-Wilcoxon test and sign test were used for non-normally distributed variables. Repeated measure analysis of variance was used for a variable measured over different periods of time to control for the baseline effect on subsequent measures.

**Results:**

Our results demonstrated that both systemic administration 30 min before and local venous pretreatment with ondansetron were equally beneficial in reducing pain during propofol injection.

**Conclusion:**

A systemic administration of ondansetron may play a role in the attenuation of propofol injection pain.

**Main Points**

• Lignocaine and ondansetron pretreatment have been found to be effective in the alleviation of propofol injection pain.

• Systemic administration of ondansetron was compared with ondansetron pretreatment in this study.

• Systemic administration of ondansetron may play a role in alleviating propofol injection pain.

## Introduction

Given its rapid onset and short duration of action, ease of titration, and benign side effect profile, propofol 2,6-di-isopropyl phenol is an extremely popular medication for inducing anaesthesia worldwide.^1^ Propofol injections, however, cause discomfort in roughly three out of five individuals, with a third of these patients report severe pain. According to several of these patients, the most unpleasant phase of the perioperative period was anaesthesia’s induction. To alleviate this pain from propofol injection, many therapies have been researched. According to a 2000 comprehensive review, the most efficient technique was venous occlusion followed by lidocaine pretreatment.^2^ However, due to the time required to apply the tourniquet, this approach is not very popular. The discomfort brought on by the injection of propofol continues to be a matter of concern and more than 100 new researches have looked into additional and alternative methods. These include novel propofol emulsions,^3,4^ modified emulsions, and microemulsion formulations,^5,6,7^ part from other drugs and interventions.

The 5-hydroxytryptamine (5-HT) antagonist ondansetron blocks sodium channels in rat brain neurons and is 15 times more potent than lidocaine on subcutaneous injection.^8^ Ondansetron is a useful alternative for the alleviation of propofol injection pain.^9,10^

Intravenous ondansetron has also been found to be an effective treatment for neuropathic pain.^11^ However, there are conflicting reports about this role of ondansetron. The local anaesthetic lidocaine has been found to alleviate the pain of propofol injection by both local anaesthetic and central analgesic effects.^12^ We designed our study to determine if systemic ondansetron was also effective in attenuating pain on propofol injection similar to ondansetron pretreatment. Our hypothesis was based on the premise that propofol injection pain is systemically induced, as suggested by Nakane and Iwama.^13^

## Methods

This double-blind randomized controlled trial was conducted after obtaining ethical approval from the Institute Ethics Committee, Indira Gandhi Institute of Medical Sciences: Sheikhpura: Patna-14 Office of the (approval no: 1077/IEC/IGIMS/2019, date: 03.10.2019). The trial was registered prospectively with the national trial registry. Before enrolment, written informed consent was obtained from all patients. This manuscript adhere to the applicable CONSORT guidelines. The study was conducted at a university hospital between February 2020 and March 2021 and is in accordance with the tenets of the Helsinki Declaration (as amended in 2013).

We included 80 patients aged 18-60 years of either gender and American Society of Anesthesiologists (ASA) class I to II scheduled for elective surgery. Exclusion criteria included patient sensitivity to ondansetron and those on concomitant analgesics, sedatives, or antiepileptic medications. Patients were randomly allocated into two groups with 40 patients in each group using a computer-generated randomization list. Sequentially numbered, opaque sealed envelopes were used to conceal the randomization sequence. The investigator and the patient were unaware of the group allocation. An independent clinician prepared the study medication.

The previous evening, all patients were orally provided alprazolam 0.5 mg and ranitidine 150 mg. On the day of surgery, no premedication was administered. A 20-gauge intravenous cannula was placed in the dorsum of both hands as soon as the patient entered the operating room, following the application of ECG, non-invasive blood pressure, and pulse oximeter monitoring. No analgesics were administered before induction. On the left upper arm, a pneumatic tourniquet was applied, and the pressure was raised to 70 mmHg to cause venous occlusion.

Patients in group S received ondansetron 4 mg (2 mL) (Ondem, Alkem Laboratoris Ltd, Mumbai, India) in saline (2 mL) intravenously over 10 s in the right hand. They were given 5 mL of the pretreatment solution (saline) intravenously 30 min later over the course of 10 s, while the venous drainage was restricted by applying a pneumatic tourniquet to the upper arm at a pressure of 70 mmHg. The occlusion was released after 2 min. Group L patients received 4 mL saline intravenously over 10 s in the right hand. After thirty minutes, patients received a 5 mL pretreatment solution (4 mg ondansetron in saline) intravenously in the left hand over a period of 10 s,^14^ while a pneumatic tourniquet (pressure raised to 70 mmHg) was applied to the upper arm to occlude venous drainage. The occlusion was released after 2 min. Thereafter, one fourth of the total calculated dose of propofol (Propofol-Lipuro, B Braun Ltd, Melsungen, Germany) stored at room temperature was administered for a period of 5 s and patients were assessed by an independent clinician for pain intensity. We questioned each patient if they found the injection to be comfortable. The verbal response was observed along with behavioral cues such as tears, facial grimacing, or arm withdrawal.^15^ The pain was graded on a scale of 0 to 3, with 0 indicating no pain, mild pain, moderate pain, and severe pain, respectively. Mean blood pressure (MAP) and heart rate (HR) were recorded immediately before the interventions and before and after propofol administration. Rescue medications in the form of atropine for bradycardia less than 50 bpm and mephenteramine for hypotension less than 20% of the baseline value were administered. After giving fentanyl, the remaining amount of propofol was used to continue the anaesthetic induction. Vecuronium was used to assist tracheal intubation, while isoflurane, nitrous oxide in oxygen, and intermittent positive pressure breathing was used to maintain anaesthesia.

### Statistical Analysis

The primary objective of this study was to determine the incidence and severity of pain on propofol injection following local or systemic administration of ondansetron. In one study, the incidence of propofol pain was 46% when patients were administered 4 mL of saline intravenously over 10 s.^15^ Another study showed a 25% incidence of pain among patients who received ondansetron 4 mg in 2 mL saline intravenously over 10 s.^9^

Based on these informations, the sample size, at 5% level of significance and 80% power to detect the difference in incidence rate between the two groups, was approximately 80, i.e., 40 in each group.

Analyses were performed using Stata version 10 (Stata Corp, College Street, Houston, Texas) and IBM SPSS 22.0 (IBM Corp. Released 2013. IBM SPSS Statistics for Windows, Version 22.0. Armonk, NY: IBM Corp). Continuous variables were checked for normality assumptions using Shapiro-Wilks test. The statistical significance level was determined as *P*<0.05. Normally distributed variables were given as mean, standard deviation, and non-normal distributed variables were expressed as median and interquartile range. Independent samples *t*-test was used for normally distributed data comparing two groups, whereas the Mann-Whitney U test was used for non-normally distributed variables. Paired sample *t*-test was used, and Wilcoxon signed-ranked tests were used to compare dependent samples. Repeated measure analysis of variance was used for variables measured over different periods.

Mauchly’s test of sphericity was used for checking the sphericity assumptions. In the case of significant violation of the sphericity assumption, Greenhouse-Geisser corrections were applied for adjusting the degree of freedom. Post-hoc comparisons between different pairs of time were done after Bonferroni corrections.

## Results

**Figure 1:**
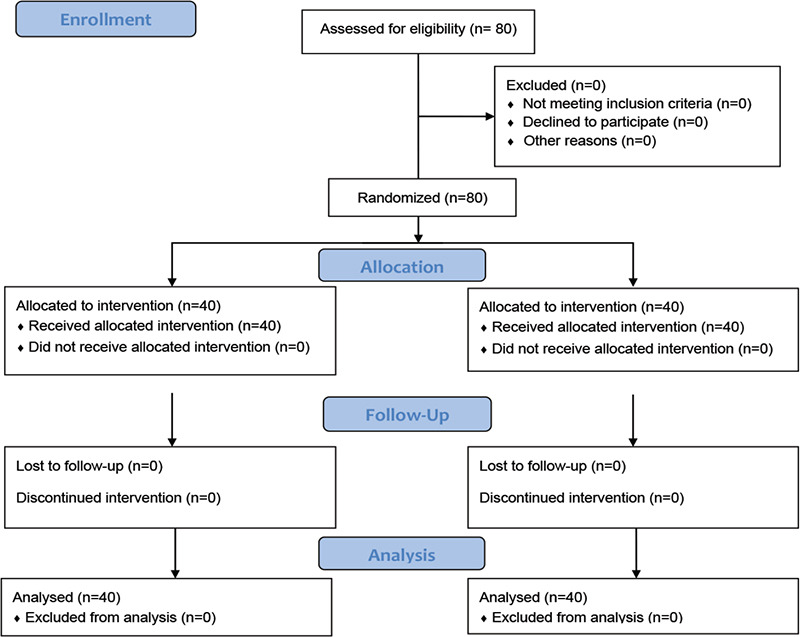
Consolidated standards of reporting trials flow diagram of participants through the study.

A total of 80 patients were included in the study and there was no dropouts (Figure 1). Table 1 displays the demographic details of the study groups. When age, gender, weight, and ASA class were compared between the groups, there was no significant difference between them. Table 2 presents the pain score measured for the patients in both groups and was found to be non-significant (*P*=0.793).

A repeated measures ANOVA was applied to test the equality of mean HR across three time points, as shown in Table 3. Mauchly’s test of sphericity indicated that the assumption of sphericity had been violated significantly (chi-square at 2 df: 136.618, *P*=0.0001). Hence Greenhouse-Geisser corrections were applied for adjusting the degree of freedom. The mean HR was significantly different across the three time points [F (1.093, 77)=25.305, *P*=0.0001)]. A post-hoc pairwise comparison using the Bonferroni correction showed a minimum change in HR between the baseline assessment and before pre-treatment assessment (mean difference =-0.138), but this was not statistically significant (*P*=1.00). However, a decrease in HR reached significance when comparing the initial assessment to post-treatment assessment (mean difference =-5.688, *P*=0.0001) and also between pre- and post-treatment assessments (mean difference =-0.5825, *P*=0.0001), respectively. Therefore, we can conclude that the results of ANOVA indicate a significant difference in HR between the two groups at various time intervals.

Repeated-measures ANOVA was applied to test the equality of mean MAP across three time points, as shown in Table 4. Mauchly’s test of sphericity indicated that the assumption of sphericity had been violated significantly (chi-square at 2 df: 98.02, *P*=0.0001). Hence Greenhouse-Geisser corrections were applied for adjusting the degree of freedom. The mean MAP was significantly different across the three time points [F (1.163, 77) =94.604, *P*=0.0001)]. A post-hoc pairwise comparison using the Bonferroni correction showed a minimum change of MAP between the baseline assessment and before pre-treatment assessment (mean difference =-0.438), but this was not statistically significant (*P*=0.706). However, a decrease in MAP reached significance when comparing the initial assessment to post-treatment assessment (mean difference =-11.40, *P*=0.0001) and also between pre- and post-treatment assessment (mean difference =-10.96, *P*=0.0001), respectively. Therefore, we can conclude that the results of ANOVA indicate a significant difference in MAP between the two groups at various time intervals.

## Discussion

Our results suggest that both systemic administration and local venous pretreatment with ondansetron were equally effective in alleviating pain on propofol injection.

Intravenous pretreatment with ondansetron has been successful in attenuating pain on the injection of propofol.^9^ A single intravenous dose of ondansetron was found to act as an analgesic for neuropathic pain, suggesting its systemic action.^11^ We planned our study to find out if systemic ondansetron was also effective in alleviating propofol injection pain similar to local ondansetron pretreatment.

Ondansetron is routinely used at our centre for the prevention of postoperative nausea and vomiting, usually in a dose of 4 mg. Based on an animal experiment, it was felt that 30 min was appropriate for ondansetron to reach the cerebrospinal fluid and exert its systemic action.^14^

In our study, 42.5% of the patients who received systemic ondansetron reported no pain on injection compared with 50% of those administered local pretreatment. Also, the incidence of moderate pain (20% versus 12.5%) and severe pain (7.5% versus 5%) was higher in the patients who were administered systemic ondansetron compared with the local ondansetron pretreatment group. However, none of these were statistically significant (*P*=0.793).

The baseline hemodynamic profile was not different in both groups and so were the hemodynamic changes following propofol administration. Patients in both groups saw a significant drop in HR and MAP as compared to their baseline values, which is a reflection of the normal effect of propofol administration.

5-HT3 receptors have been found to play a role in spinal pain transmission and endogenous pain suppression. They are expressed in the monoaminergic descending inhibitory system, certain brain regions, autonomic afferents, peripheral nerve terminals, and other tissues. When spinal 5-HT3 receptors in the dorsal horn are stimulated, they produce an antinociceptive response probably due to the release of GABA and subsequent activation of the descending inhibitory system.^16^ The 5-HT3 receptor antagonists interrupt this antinociceptive effect.

Skin, mucous membrane, and venous intima get irritated by propofol, which is chemically phenol. A high aqueous free propofol concentration has been implicated in causing injection pain.^17^ Nakane and Iwama,^13^ proposed a systemic mechanism for this pain, whereas the dissociation of propofol activates the plasma kallikrein-kinin system, releasing bradykinin and causing pain. This was substantiated when it was observed that centrally acting analgesics like tramadol, ketamine and non-steroidal anti-inflammatory drugs like flurbiprofen also alleviated this pain.^12^ Non-selective ligand-gated cation channels such as transient receptor potential (TRP), ankyrin 1, and TRP vanilloid 1 have been found to mediate release of neuropeptides and produce propofol-induced pain.^18^

We used a propofol formulation containing medium chain triglyceride because it has a reduced concentration of free propofol in the aqueous phase and is known to cause lesser pain on injection.^19^

In animal models of nerve damage, it has been hypothesized that intrathecal injection of 5HT-3 receptor antagonists such as ondansetron reduces mechanical and thermal hypersensitivity.^20,21^ Serotonin plays a crucial role in the endogenous analgesia process. Serotonergic neural regulation that descends to the spinal cord from the rostral ventromedial medulla reduces neuronal activity and hypersensitivity and aids in analgesia.^22^ This has been linked to the serotonin activity on the G-protein coupled 5HT-1 and 5HT-7 subtypes of serotonin receptors.^23^ G-protein coupled receptors also play some role in the attenuation of propofol-induced pain.^9^ However, there are certain limitations to our study. Due to ethical concerns, a placebo group was not included in our study. Estimation of cerebrospinal fluid and serum levels of ondansetron could not be performed for logistic reasons.

## Conclusion

The findings that systemic administration of ondansetron may play a role in alleviating propofol injection pain can be a basis for further research into its use as an analgesic in pain models other than neuropathic.

## Figures and Tables

**Table 1:**
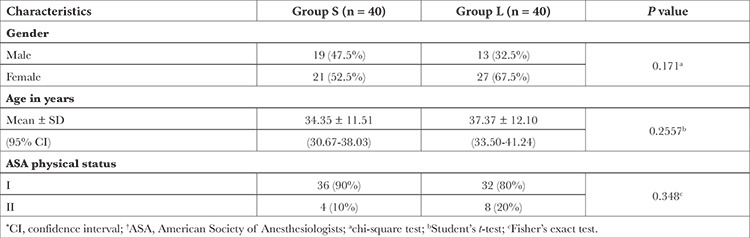
Comparison of Demographic Profile

**Table 2:**
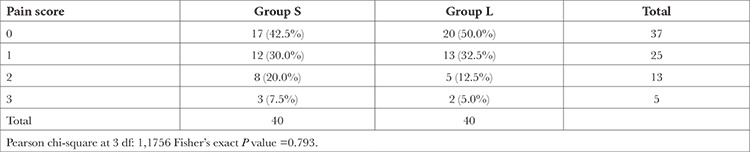
Comparison of Pain Scores Between the Groups

**Table 3:**
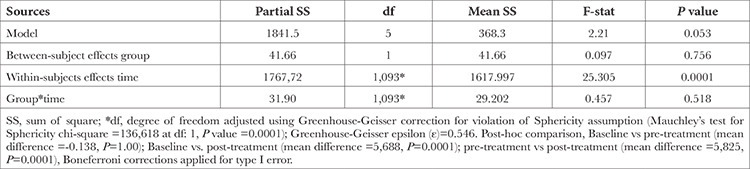
Repeated Measure ANOVA of Heart Rate Over Time

**Table 4:**
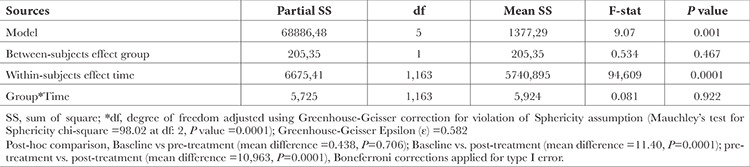
Repeated Measure ANOVA of MAP Over Time
